# Quantifying the Effect of Xiluodu Reservoir on the Temperature of the Surrounding Mountains

**DOI:** 10.1029/2019GH000242

**Published:** 2020-05-01

**Authors:** D.C. Wang, J.Y. Liu, Y. Huang, X.W. Duan, X. Wang, X. Zhang, Z.C. Sun, J.H. Chen, W. Zhang

**Affiliations:** ^1^ School of Geology and Geomatics Tianjin Chengjian University Tianjin China; ^2^ Institute of International Rivers and Eco‐Security Yunnan University Kunming China; ^3^ Yunnan Key Laboratory of International Rivers and Trans‐Boundary Eco‐Security Yunnan University Kunming China

**Keywords:** effect threshold, factor analysis, partial correlation analysis, temperature differences, Xiluodu Reservoir

## Abstract

To quantitatively determine the effect of Xiluodu Reservoir on the temperature of the surrounding mountains, the temperature differences between various locations and the reservoir were calculated based on Landsat 8 thermal infrared sensor (TIRS) data. Elevation, slope, aspect, normalized difference vegetation index (NDVI), and visual field were selected as the impact factors, and the most significant grid size used to explore the effect of reservoir on the surrounding mountains was determined by spatial analysis and partial correlation analysis. The effect of the Xiluodu Reservoir on the surrounding mountains' temperature was then quantitatively studied while accounting for the effect of water surface width on temperature. The results are summarized as follows. The most significant grid size for determining the influence of Xiluodu Reservoir on the surrounding mountains' temperature is 90 m. The effect range threshold of the entire reservoir on the temperature of the surrounding mountains is approximately 600 m, and the partial correlation coefficient in each buffer area decreases gradually with increasing distance from the reservoir. The effect threshold of the reservoir on the temperature of the surrounding mountains is approximately 1,500 m in the head area with a water surface width approximately 1,000 m, but it is negligible in the tributary area where the width is approximately 60 m.

## Key Points

- Elevation, slope, aspect, normalized difference vegetation index (NDVI), and visual field were selected as the impact factors
- To overcome the scale‐dependence of LST, the most significant grid size was determined by spatial analysis and partial correlation analysis
- Study the effect of reservoir on the surrounding mountains' temperature while accounting for the effect of water surface width

## 1 Introduction

As the typical area with a dry‐hot valley climate characteristic of the special ecosystem type in southwest China (Duan et al., 2015), the lower reaches of the Jinsha River faces many ecological problems due to its complex terrain and geology along with human activities (Chen et al., 2003; Miao et al., 2011; Yang et al., 2016). As the first phase of Jinsha River hydropower development, the Xiluodu Hydropower Station is the second largest in China and the third largest in the world with the area of more than 140 km^2^. It also has the capabilities of controlling floods, sediment retaining, and improving downstream shipping conditions with the huge installed and regulation capacities of 12,600 MW and 6.46 billion m^3^, respectively. The reservoir can affect heat transmission to the surrounding environment because the water body in it has a large heat capacity and thermal inertia along with low heat conduction and thermal emissivity. Thus, the reservoir cannot be neglected when considering the regional ecosystem balance, the heat island effect, and the local microclimate (Xu & Yue, 2008; Yu et al., 2015; Miao et al., 2016; Guo et al., 2019). For this reason, quantifying the effect threshold of Xiluodu Reservoir on the temperature of the surrounding mountains is important for protecting the natural environment and biodiversity of the area and ensuring the prudent use of reservoir resources (Kong et al., 2015; Null et al., 2013).

Many studies have been reported on both domestic and international water bodies, it is important to understand the mechanism of regional‐scale climate change with respect to natural landscapes such as water bodies, particularly in relation to the increasing severity of the urban heat island effect as a result of urbanization (Huang & Lu, 2015; Heinl et al., 2015; Wu et al., 2017). Bokaie et al. (2016) found that water bodies can effectively mitigate the intensity and spreading rate of urban heat islands. Yue and Xu (2013) and Sun et al. (2012) reported that water bodies were cooler than their surrounding environment. Water bodies, which have high specific heat capacity, can affect the rate of temperature change through state change, which affect the latent heat exchange capacity.

The cooling effects of different water bodies will differ based on the environment, which affects the cooling strength and efficiency. The studies mentioned above were mostly qualitative investigations of the cooling effects of water bodies on the surrounding environments (Chun & Guldmann, 2014; Zhang et al., 2016; Wu et al., 2014). However, quantitative studies of water temperature regulation have begun to receive more attention, including studies on the effects of waterbody shape and size on cooling efficiency and the extent to which water can regulate temperature (i.e., threshold study) (Wu et al., 2017; Broadbent et al., 2017; Hicham et al., 2016; Cao et al., 2011; Chang et al., 2007; Chen et al., 2013; Miao et al., 2015). Ghosh and Das (2018) found there was a close relationship between the cooling effect of the water body and its shape index value, which the water body can reduce the temperature within 70 m by more than 1 °C. Compared to commercial areas, Li et al. (2008) found a clear negative linear relationship (correlation coefficient = −0.72) between the proportion of water surface and land surface temperature (LST) in Dongguan City. The effective range of the river's effect on the urban heat environment was approximately 200 m, and the cooling effect varied with river width. Sun and Chen (2012) studied the water body in Beijing six rings and found that the cooling island range of 116 water bodies was 100 m or less, and a threshold in the width of water body was needed to ensure its cooling effect. Qiu et al. (2017) found urban water bodies and urban green spaces can relieve the urban heat island up to 0.9 and 1.57 °C, respectively, which shows the cooling effect of later is better than that of former. It can be seen that, as an aspect that cannot be ignored in quantitative research of water bodies, threshold research can often point out the maximum efficiency and the maximum range of water cold island in a particular research area which can provide a definite numerical value for the planners or the architects to make reasonable estimation and planning.

However, most studies have focused on relatively flat urban areas, whereas few have investigated environments with complex terrain, where studying the effect of water on the surrounding environment requires the consideration of additional factors. In the mountain city of Chongqing, Li and Yu (2014) collected temperature data around three lakes and found that the lake's maximum cold island effect was 2.9 °C, and it was affected by the lake area and shape, the comprehensive canopy temperature, and the distance from the lake. Some studies demonstrated that LST can affect the surface energy balance in mountainous areas, and it was influenced by many factors, including terrain, altitude, incident radiation, atmospheric processes, soil moisture distribution, and the complex interactions between land cover and vegetation types (Bertoldi et al., 2010; Hamada et al., 2013; Zhao et al., 2016). While the above studies only considered some of the factors affecting the surrounding temperature, most of these factors will be comprehensively considered in this study.

Although the LST retrieved from remote sensing images is not equivalent to the actual near‐surface air temperature, they are highly correlated with each other (Ogashawara et al., 2019; Bechtel et al., 2012; Cho & Suh, 2013; Zhao et al., 2014; Wang et al., 2016; Weng, 2009); thus, the actual near‐surface air temperature was replaced by LST in studies. And most of the above temperature studies were based on MODIS remote sensing images (Schwarz et al., 2011). Compared with MODIS products (MYD11A2), the new thermal infrared sensor (TIRS) of Landsat 8 contains two thermal infrared bands of 10 and 11 with higher spatial resolution (100 m than that of MYD11A2, 1,000 m); therefore, the band 10 was used in temperature retrieval in this study based on Landsat 8 TIRS images.

In this study, Xiluodu Reservoir was selected as the study area not only because of its large scale and large volume but also its location in the complex mountain environment. The temperature differences between various locations and the reservoir were calculated based on Landsat 8 TIRS data to quantitatively determine the effect of Xiluodu Reservoir on the temperature of the surrounding mountains. Spatial analysis and partial correlation analysis were utilized to determine the most significant grid size. Elevation, slope, aspect, normalized difference vegetation index (NDVI), and visual field were selected as the impact factors to quantitatively and comprehensively evaluate the effect of the Xiluodu Reservoir on the surrounding mountains' temperature, taking the width of water surface into consideration.

## 2 Materials and Methods

### 2.1 Study Area

**Figure 1:**
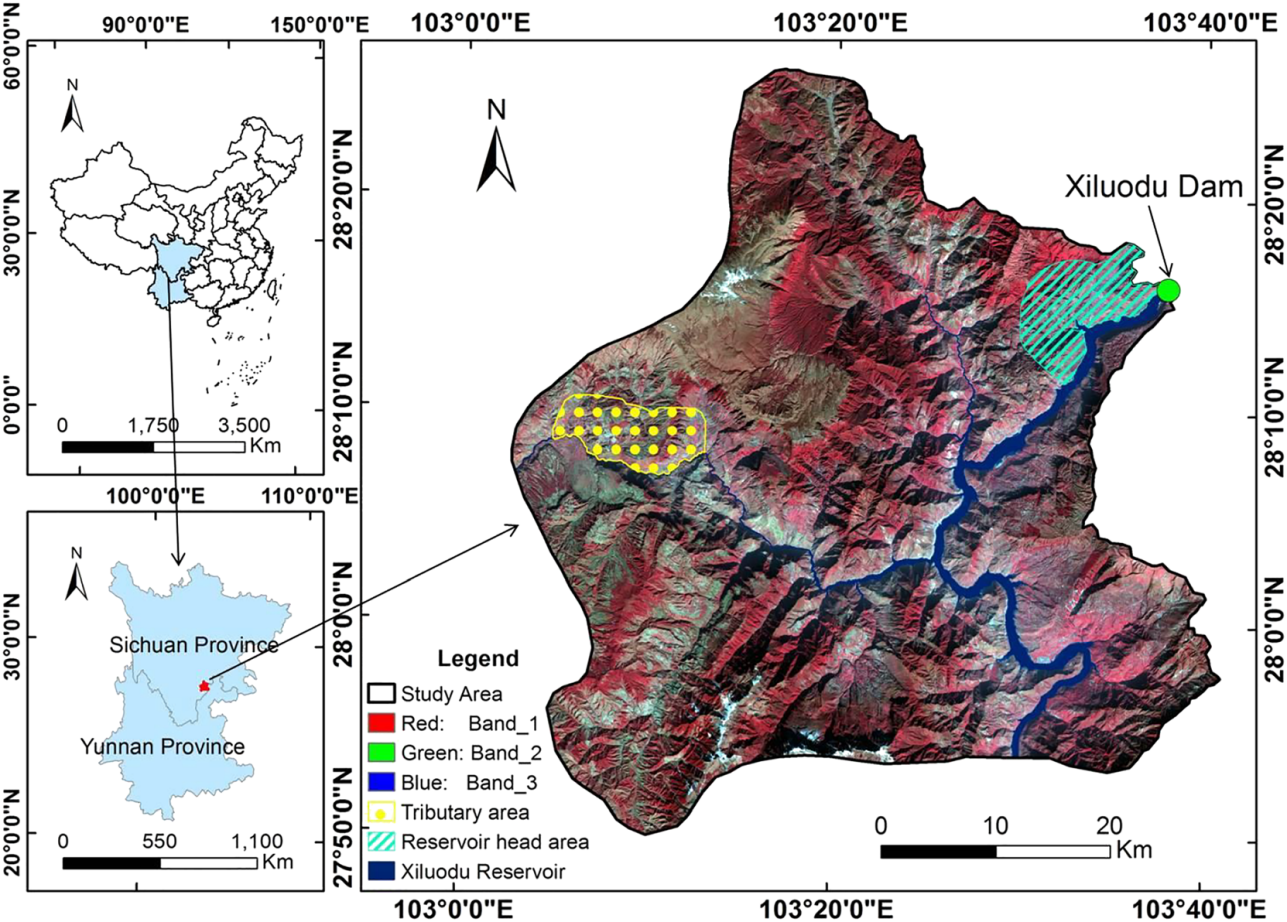
Location of the study area.

As the third largest hydropower station in the world, the Xiluodu Hydropower Station extends from the slope areas of the Qinghai‐Tibet and Yunnan‐Guizhou Plateaus to Sichuan Basin. The total installed capacity of Xiluodu Reservoir is 12,600 MW, and the regulating and flood control capacities are 6.46 and 4.65 billion m^3^, respectively. In addition to being central to power transmission in China from west to east, the Xiluodu Hydropower Station is important for allocating energy, improving the environment and promoting sustainable development. The head area of the Xiluodu Reservoir from 27°50′–28°20′N and 103°00′–103°40′E (Figure 1) was selected as the study area. Figure 1 shows the spatial extent, geographic location and corresponding digital elevation model (DEM) data of the study area.

The study area has a dry‐hot valley climate with a cold and humid climate in the alpine region, and significant variations in climate are observed in the study area. The regional vegetation belongs to subtropical evergreen broadleaf forest, with the climate and soil distribution has a vertical distribution characteristic. And the terrain in the study area is undulating in the view of terrain features.

**Figure 2:**
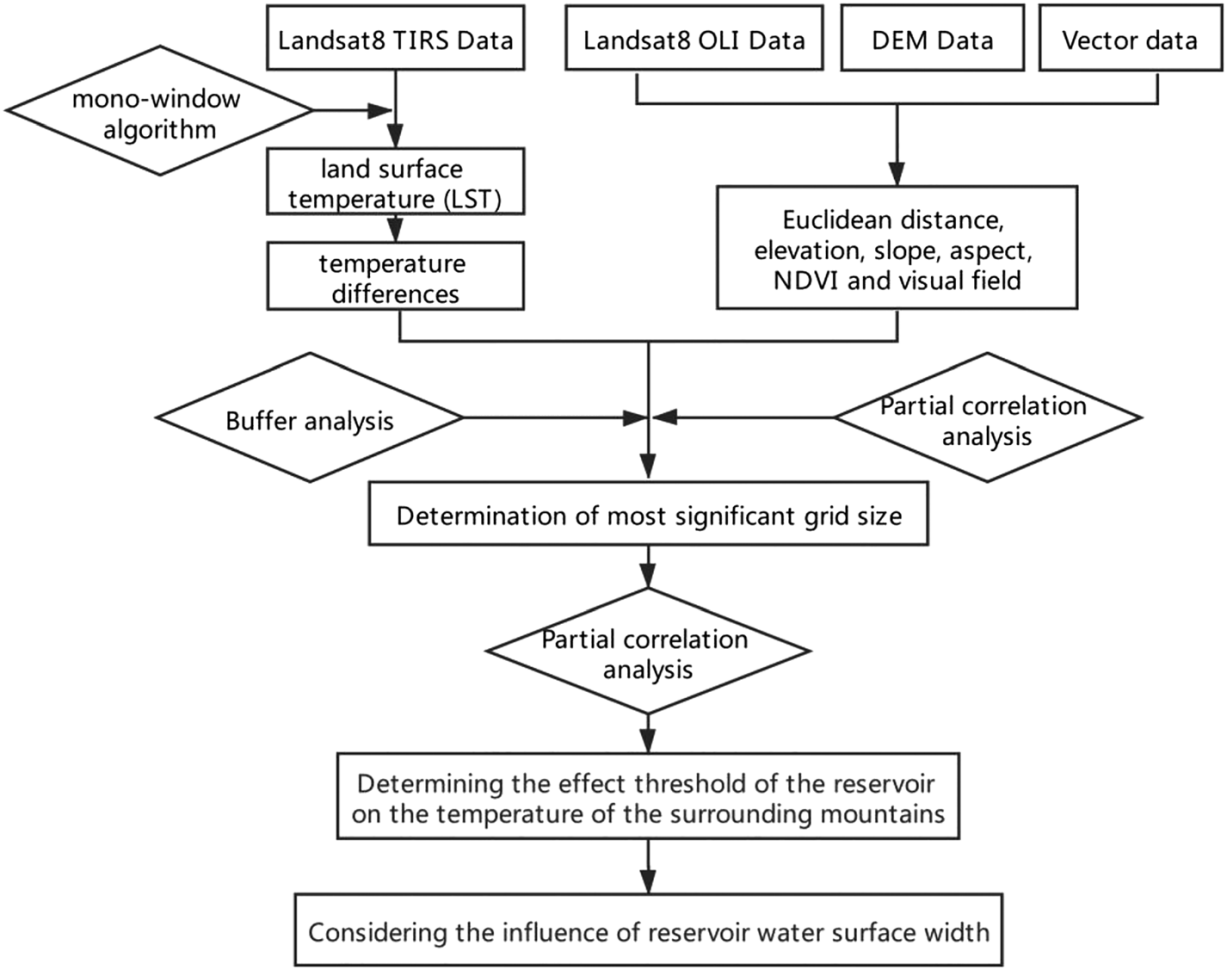
The technical flowchart for this study.

To quantitatively determine the effect of Xiluodu Reservoir on the temperature of the surrounding mountains, the following steps were taken in this study (Figure 2): first, retrieving LST and calculating the temperature difference based on remote sensing image; second, determining and obtaining the spatial distribution data of image factors; third, determining the most significant grid size for this study; and fourth, quantitatively studying the effect of Xiluodu Reservoir on the temperature of the surrounding mountains, considering the effect of water surface width on temperature at the same time.

### 2.2 LST Retrieval and Calculation of Temperature Difference

With the rapid development of remote sensing technology, the use of thermal infrared bands in remote sensing data has become a convenient way to retrieve LST. At present, LST retrieval algorithms based on remote sensing data include the radiative transfer equation method (Wu et al., 2016), mono‐window algorithm (Qin et al., 2001; Wang et al., 2015), split‐window algorithm (Du et al., 2015; Rozenstein et al., 2014), and multichannel and multi‐angle algorithm (Sobrino et al., 1996). Based on previous research results, the mono‐window algorithm is simple operation, widely applicably and precise. According to the U.S. Geological Survey, compared with the great uncertain band 11, the Landsat 8 TIRS band 10 data should be used as the single‐band thermal infrared data (Zhang et al., 2009; Zhao et al., 2014). Therefore, the LST of the study area was retrieved on Landsat 8 TIRS band 10 data based on the mono‐window algorithm (Qin et al., 2003).

The calculation principle of the mono‐window algorithm can be expressed as (Wang et al., 2015; Wu et al., 2016)

(1) $$T_{S}=\left\{a_{10}\left(1-C_{10}-D_{10}\right)+\left[b_{10}\left(1-C_{10}-D_{10}\right)+C_{10}+D_{10}\right]T_{10}-D_{10}T_{a}\right\}/C_{10},$$

where T_S_ is the LST retrieved from the Landsat 8 TIRS band 10 data; a_10_ and b_10_ are constants that depend on the range of surface temperature (a_10_ = −55.4276 and b_10_ = 0.4086) (Wang et al., 2015; Zhao et al., 2016); C_10_ and D_10_ are internal parameters. T_10_ is the brightness temperature of Landsat 8 TIRS band 10; and T_a_ is the effective average atmospheric temperature. The calculation of parameters in equation (1 refers to other scholars (Jiang et al., 2015; Wu et al., 2016; Zhao et al., 2016).

The concrete process of LST retrieval based on Landsat 8 TIRS band 10 data using the mono‐window algorithm is summarized as follows: (1) convert the digital number to thermal spectral radiance; (2) according to the Planck theorem, convert the resulting thermal spectral radiance to the brightness temperature; (3) according to the mixed model developed by Qin et al. (Qin et al., 2003), calculate the ground emissivity; and (4) calculate LST using the ground emissivity and other parameters.

This paper aimed to clarify the effect of the reservoir on the temperature of the surrounding mountains. Thus, the temperature differences between various locations and the reservoir (*∆*LST) were calculated as follows:

(2) $$\mathrm{∆LST}=\mathrm{LST}-{\mathrm{LST}}_{0},$$

where LST is the LST of the study area and LST_0_ is the temperature of the reservoir.

### 2.3 Factor Analysis of the Mountains Temperature

Because of the complex terrain in the mountainous part of the study area, the factors affecting its temperature distribution are different. From a climatic point of view, the factors can be summarized as macro‐geographical conditions, elevation, topography, and underlying surface properties (Ogashawara & Bastos, 2012; Papangelis et al., 2012; Onishi et al., 2010). Taking into account the particularity of the mountainous terrain, the ground distance between two points on the ground cannot represent the true path affected by the temperature between them. Therefore, Euclidean distance (i.e., the shortest straight‐line distance from each point in the study area to the reservoir) was used as an impact factor. Similarly, each point in the study area participated in the calculation should be visible to the reservoir, so the visible field of the study area to the reservoir were selected as another impact factor. Therefore, the Euclidean distance from the reservoir, elevation, slope, aspect, NDVI, and visual field of the study area were selected as impact factors.

To further clarify the effects of the selected impact factors on the temperature difference, the elevation, slope, aspect, and NDVI of the study area were divided into different grades. Elevation was divided into 400 m zones up to 4,000 m. For slope, when slope < 45°, 5° intervals were used, and one group with slope > 45° was created. Aspect was classified according to the conventional eight‐aspect classification method: East (67.5°–112.5°), Southeast (112.5°–157.5°), South (157.5°–202.5°), Southwest (202.5°–247.5°), West (247.5°–292.5°), Northwest (292.5°–337.5°), North (0°–22.5°, 337.5°–360°), and Northeast (22.5°–67.5°) (Dan et al., 2009; Yang et al., 2014). NDVI was divided into nine grades based on the available data: NDVI < 0; seven groups with NDVI from 0 to 0.35 in intervals of 0.05; and NDVI > 0.35. Finally, the relationships between the impact factors and the temperature difference were determined by calculating the average of them in each grade.

Based on the DEM data, the Euclidean distance, elevation, slope, aspect, and visual field of the study area were calculated using ArcGIS 10.2. NDVI was calculated with ENVI 5.2.

### 2.4 Determination of Most Significant Grid Size

Numerous studies have confirmed that the pattern of geography research objects and the occurrence of the process, time and space distribution, mutual coupling, and other characteristics were scale dependent. Thus, the characteristics of these research objects have the characteristics of space‐time scale, and LST in the time and space range also showed great heterogeneity (Zhao et al., 2016; Zhao & Xie, 2010). It can be deduced that the distribution of temperature difference in space is also scale dependent; that is, it changes with grid size. Thus, an appropriate scale is needed to determine the effect threshold of the Xiluodu Reservoir on the temperature of the surrounding mountains (Weng & Hu, 2011).

Considering that the terrain of the study area is mountainous, the representation of the calculated factors is closely related to the grid size. The rougher the grid is, the less representative the average of the various factors calculated in each grid is. Therefore, considering the operability of the data and the size of the research area, based on the original resolution of 30 m, five grid sizes were tested: 30, 60, 90, 120, and 150 m. For each grid size, six different buffer zones were established with different distances from the reservoir: 300, 600, 900, 1,200, 1,500, and 1,800 m. After calculating the average for each impact factor in each grid size, the most significant grid size was determined by comparing the partial correlation coefficients for the relationship between temperature difference and Euclidean distance within each buffer zone.

### 2.5 Partial Correlation Analysis

When discussing the effect threshold of the Xiluodu Reservoir on the temperature of the surrounding mountains, the relationships between the temperature difference and the different impact factors are complicated and difficult to determine because the temperature difference is affected by many factors. Therefore, partial correlation analysis is necessary to truly reflect the relationships between the temperature difference and the impact factors (Dan et al., 2009). Thus, partial correlation analysis was carried out using four impact factors (elevation, slope, aspect, and NDVI) as the control variables within the scope of visualization. Subsequently, the partial correlation coefficient between the temperature difference and Euclidean distance in each buffer zone was calculated to determine the effect threshold of the reservoir on the temperature of the surrounding mountains. Under the most significant grid size, the distance between each buffer is set to 100 m from 100 to 1,000 m.

In this study, partial correlation analysis was carried out using SPSS 22.0 software. Because of the large scope of the study area, partial correlation analysis was applied separately to the area surrounded by the main reservoir area and the tributary area.

### 2.6 Effect of Water Surface Width on Temperature

Because the water surface width of the reservoir varies in the study area, the effect of water surface width on the surrounding mountains' temperature was evaluated (Yue & Xu, 2013; Li et al., 2008). Two unilateral buffers with the lengths of approximately 15,000 m and the width of 5,000 m from the edge of the reservoir were intercepted in the reservoir head area and tributary area, respectively, as shown in Figure 4. The water surface width of the reservoir head area was approximately 1,000 m, showing the north south distribution trend. The water surface width in the tributary region was narrow (~60 m), showing the east west distribution trend. Partial correlation analysis and buffer analysis were carried out on the two regions to determine the effect of water surface width on the surrounding temperature.

## 3 Data

Landsat 8 TIRS images acquired on 8 December 2016 and 25 January 2017 with less cloud cover were used to retrieve LST, which were downloaded from the Geographic Spatial Data Cloud website (http://www.gscloud.cn/). PATH/ROW was 129/41. The average of the retrieved temperature was used as the basic temperature data of the study area.

The historical temperature data of 8 December 2016 and 25 January 2017 were downloaded from a website (http://www.tianqihoubao.com/lishi/leibo.html). DEM data of the study area were downloaded from the Geographic Spatial Data Cloud website (http://www.gscloud.cn/) and used to extract the temperature impact factors.

## 4 Results

### 4.1 Distributions of LST and Temperature Difference

**Figure 3:**
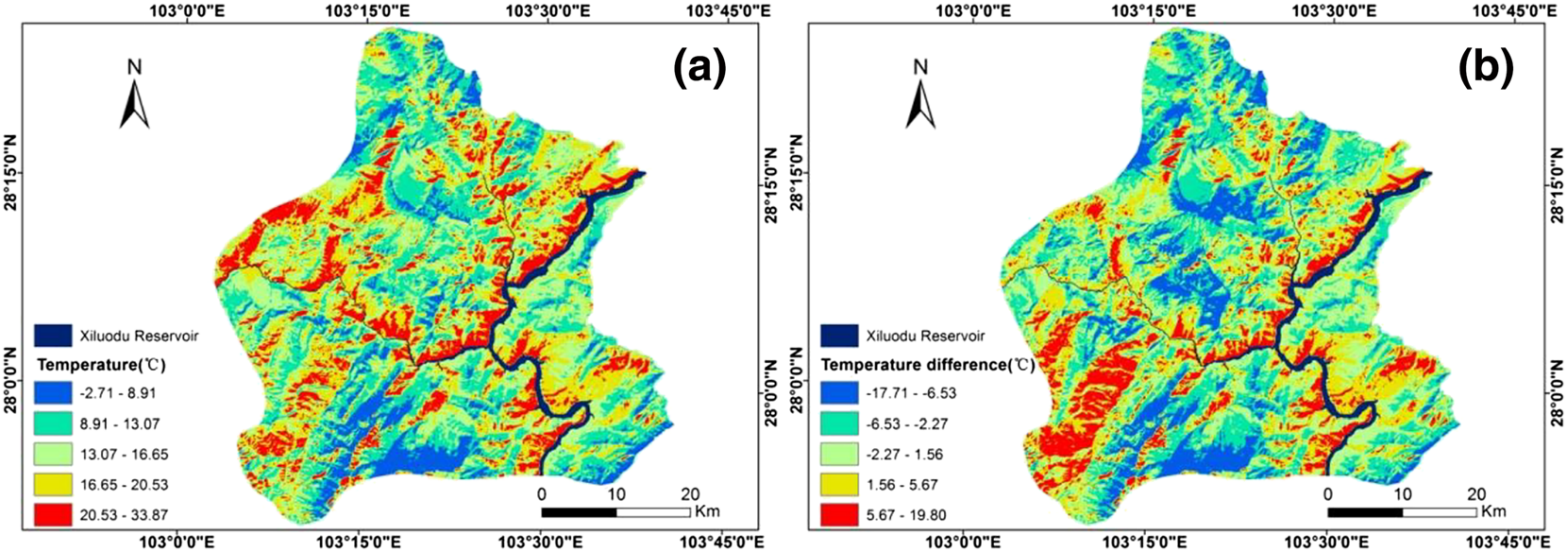
Spatial distributions of LST (a) and temperature difference between the study area and the Xiluodu Reservoir (b).

The LST of the study area was retrieved using the mono‐window algorithm (Figure 2a), and the temperature differences between different points in the study area and the Xiluodu Reservoir were calculated (Figure 3b).

To more intuitively show the differences in the spatial distributions of LST and temperature difference, the natural breaks method was applied, and the difference in color distribution reflects the difference of the temperature values distribution in the study area. And the highest and lowest LST values were 33.87 and −2.71 °C, respectively. In general, the temperature in the study area decreases as the distance from the reservoir increases. In addition, the LST distribution is clearly different on the two sides of the reservoir, and a region of high temperature extends along the valley. The range of temperature difference in the study area is relatively large, with maximum and minimum temperature differences of 19.80 and −17.71 °C, respectively. No clear trend is observed in the distribution of temperature difference, although some relatively large temperature differences (greater than 5.67 °C) are distributed in the southwest of the study area.

### 4.2 Analysis of Factors Affecting Temperature

**Figure 4:**
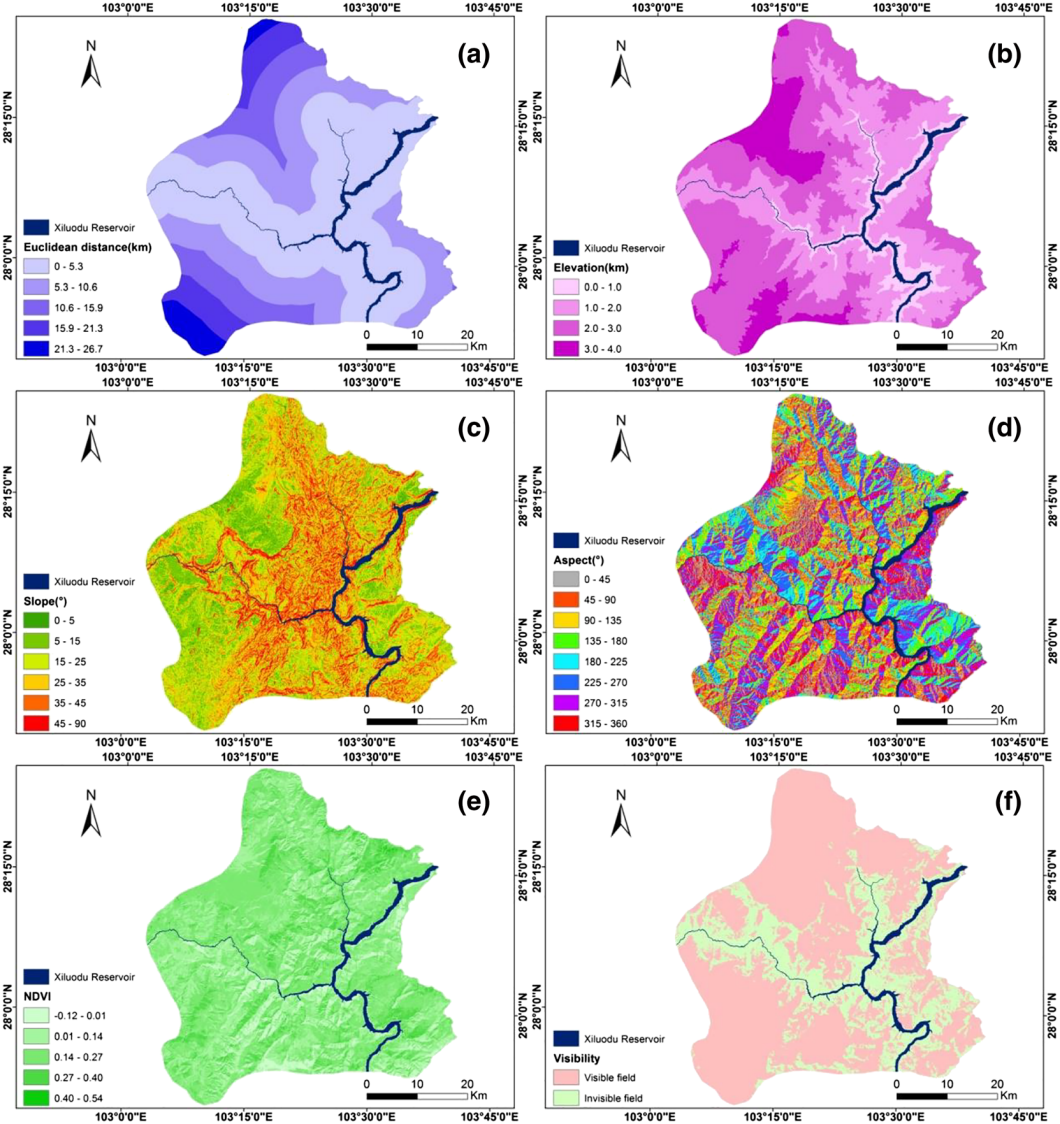
Spatial distributions of Euclidean distance (a), elevation (b), slope (c), aspect (d), NDVI (e), and visual field (f).

Based on the remote sensing images and DEM data of the study area, the Euclidean distance, elevation, slope, aspect, NDVI, and visual field of the study area are calculated and shown in Figure 4.

Because the Euclidean distance represents the shortest straight‐line distance from each point in the study area to the reservoir, the spatial distribution of Euclidean distance is a circular distribution consistent with the location of the reservoir (Figure 4a). The elevation of the entire study area is below 4 km. Because of the valley terrain, the elevation of the study area increases with increasing distance from the reservoir. Areas of relatively high elevation are primarily distributed in the northwestern and southwestern regions of the study area (Figure 4b). The slope is highest (greater than 45°) in the middle of the study area, whereas the slope in the areas far from the reservoir is relatively lower (less than 25°; Figure 4c). The spatial distribution of aspect also shows variations related to the terrain. The valley around the reservoir area shows a clear distribution of sunny and shady slopes. The mountain aspect determines the duration of sunshine and the intensity of solar radiation, which is closely related to the vegetation condition (Figure 4d). The spatial distribution of NDVI indicates that the vegetation coverage in the study area is poor (Figure 4e). Figure 4f shows the visible field of the study area to the reservoir.

Previous studies have shown that the surface temperature is correlated with elevation, slope, aspect and NDVI.

**Figure 5:**
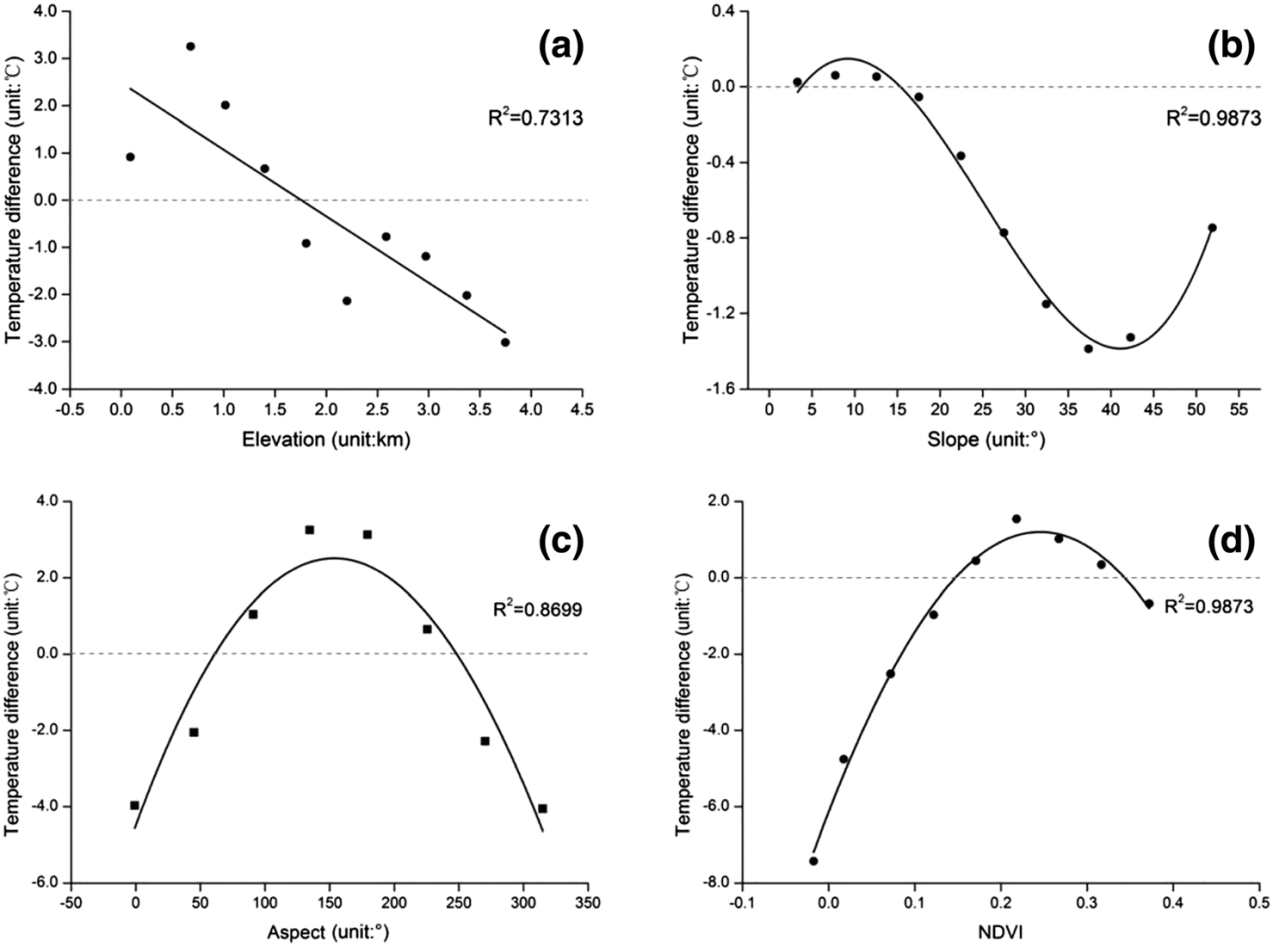
The variation in temperature difference with elevation (a), slope (b), aspect (c), and NDVI (d).

Correlation analysis indicated that temperature difference is significantly linear correlated with elevation at the level of *P* < 0.01, with coefficients of −0.855 (Figure 5a), indicating a highly relevant relationship. While a significant nonlinear correlation were founded between the temperature difference with slope (Figure 5b), aspect (Figure 5c), and NDVI (Figure 5d), respectively. Correlation analysis results indicate that these factors can affect the difference in temperature between the reservoir and the surrounding environment. Thus, it is necessary to use partial correlation analysis to determine the effect threshold of the reservoir on the temperature of the surrounding mountains.

### 4.3 Determination of Most Significant Grid Size

Partial correlation analysis results of the relationship between temperature difference and Euclidean distance for different grid sizes are shown in Table 1.

**Table 1:** Partial Correlation Analysis Results of the Relationship Between Temperature Difference and Euclidean Distance for Different Grid Sizes

Grid size	0–300 m	0–600 m	0–900 m	0–1,200 m	0–1,500 m	0–1,800 m
30 m	0.472	0.333	0.208	0.109	0.026	−0.064
60 m	0.453	0.313	0.189	0.092	0.011	−0.076
90 m	**0.475**	0.334	0.210	0.111	**0.029**	−**0.063**
120 m	0.455	0.311	0.193	0.094	0.014	−0.073
150 m	0.473	**0.335**	**0.212**	**0.115**	0.027	−0.066

*Note*. The bold numbers indicate the maximum partial correlation coefficients within each buffer area at different grid sizes. The temperature difference is significantly correlated with Euclidean distance in each buffer area at different grid sizes (*P* < 0.05). All the degrees of freedom corresponding to the partial correlation coefficients meet the statistical requirements.

As shown in Table 1, within a distance of 300 m from the reservoir, the partial correlation coefficient between the temperature difference and Euclidean distance is the largest (0.475) at the grid size of 90 m. In the other three buffer ranges (within 600, 900, and 1,200 m of the reservoir), the partial correlation coefficients between the temperature difference and Euclidean distance are largest for the grid size of 150 m (0.335, 0.212, and 0.115, respectively). The partial correlation coefficients within 1,500 and 1,800 m of the reservoir are largest (0.029 and −0.063) at the grid size of 90 m. These results indicate that the partial correlation coefficients calculated for each buffer region are relatively large at the grid size of 90 and 150 m (i.e., the partial correlation is the most obvious at this two grid size). Considering that terrain of the study area is primarily mountainous, the smaller the grid, the more representative the average of the various factors calculated in each grid. Thus, 90 m was selected as the most significant grid size for further analysis.

### 4.4 Results of Partial Correlation Analysis

Under the most significant grid size of 90 m, correlation and partial correlation coefficients for the relationship between temperature difference and Euclidean distance are calculated and shown in Table 2.

**Table 2:** Correlation and Partial Correlation Coefficients for the Relationship Between Temperature Difference and Euclidean Distance at the Most Significant Grid Size of 90 m

Distance from the reservoir	Pearson correlation	Partial correlation
0–100 m	0.464	0.476
0–200 m	0.460	0.497
0–300 m	0.421	0.475
0–400 m	0.368	0.431
0–500 m	0.324	0.380
0–600 m	0.292	0.334
0–700 m	0.271	0.292
0–800 m	0.255	0.251
0–900 m	0.239	0.210
0–1,000 m	0.229	0.174

*Note*. The temperature difference is significantly correlated with Euclidean distance in each buffer area at the most significant grid size of 90 m (*P* < 0.05).

From Table 2, the correlation and partial correlation coefficients for the relationship between temperature difference and Euclidean distance in each buffer zone decrease with distance from the reservoir at the most significant grid size of 90 m. The difference between the correlation analysis and the partial correlation analysis can be clearly seen by comparing the coefficients, which is attributed to the influence of factors considered in the partial correlation analysis. This indicates that the relationship between temperature difference and Euclidean distance is more accurately described by partial correlation analysis.

The maximum partial correlation coefficient is 0.497 in the buffer range of 0–600 m, whereas the minimum value is 0.174 when the buffer range is within 3,000 m (Table 2); however, the temperature difference is significantly correlated (*P* < 0.05) with Euclidean distance in each buffer area. Based on the statistical theory, the correlations were classified into four grades: weakly related or not related ([0, 0.3]), low related ([0.3, 0.5]), medium related ([0.5, 0.8]), and high related ([0.8, 1.0]). Then, the effect threshold of the reservoir on the temperature of the surrounding mountains is around 600 m with the partial correlation coefficient is 0.334.

### 4.5 Influence of Water Surface Width on Temperature

Partial correlation analysis and buffer analysis were carried out on the reservoir head area and tributary area, respectively. And the results of the comparative analysis are shown in Table 3.

**Table 3:** Partial Correlation Analysis Results for the Relationship Between Temperature Difference and Euclidean Distance of the Reservoir Head Area and Tributary Area

Distance from the reservoir	Reservoir head area		Tributary area	
	*R*	*P*	*R*	*P*
0–300 m	0.519	0.000	0.050	0.225
0–600 m	0.491	0.000	−0.131	0.000
0–900 m	0.472	0.000	−0.240	0.000
0–1,200 m	0.428	0.000	−0.335	0.000
0–1,500 m	0.370	0.000	−0.339	0.000
0–1,800 m	0.278	0.000	−0.273	0.000
0–2,100 m	0.142	0.000	−0.228	0.000
0–2,400 m	0.001	0.000	−0.250	0.000
0–2,700 m	−0.151	0.000	−0.276	0.000
0–3,000 m	−0.219	0.000	−0.294	0.000

*Note*. *R* is the partial correlation coefficient, and *P* is the significance level (uncorrelated probability, *P* < 0.05 indicates significantly correlated).

Table 3 indicates that water surface width has an obvious effect on temperature. In the reservoir head area, the partial correlation coefficient between the temperature difference and Euclidean distance in each buffer zone is larger than that of the entire study area. The partial correlation coefficient in each buffer zone decreases gradually with increasing distance from the reservoir, and the maximum partial correlation coefficient is 0.519 in zone of 0–300 m, and the minimum partial correlation coefficient is −0.219 in the zone of 0–3,000 m. The temperature difference is significantly correlated (*P* < 0.05) with Euclidean distance in each buffer area. Similarly, according to the statistical theory, the effect threshold of the reservoir head area with the width of about 1,000 m on the temperature of the surrounding mountains is about 1,500 m or so. In contrast, the partial correlation coefficient between the temperature difference and Euclidean distance in each buffer region is generally low in the tributary area; the maximum coefficient is 0.050 in the buffer range of 0–300 m but does not pass the 95% significance test. Although the partial correlation coefficients indicate that the temperature difference is significantly correlated with Euclidean distance in the buffer area outside of 300 m, the partial correlation coefficients are less than 0.3 (indicating weakly related or not related). In summary, the effect of the tributary area, where the water surface width is approximately 60 m, on the temperature of the surrounding mountains is very weak.

## 5 Discussion

In mountainous areas, the spatial distribution of LST was consistent with the mountain direction, and higher altitude is associated with lower temperature. The temperature of the Jialing River valley is difficult to reduce because of its low altitude (Dan et al., 2009). The results of this paper support the above findings. In the study area, the high‐temperature regions are distributed primarily near the reservoir and along the valley. In flat areas, the effect of the reservoir on temperature decreases as the distance from the reservoir increases; accordingly, the temperature difference increases with increasing distance from the reservoir. However, this trend is not obvious in the mountainous parts of the study area because the LST in mountainous areas is influenced by a variety of factors.

The effects of elevation, slope, aspect, and NDVI on the temperature difference were analyzed. The temperature difference has a negative linear relationship with elevation because elevation directly affects the heat exchange efficiency between the land surface and air in the mountainous area, resulting in a significant difference in temperature. A previous study found a negative linear relationship between temperature and slope (Zhao et al., 2016); however, a significant nonlinear relationship between temperature difference and slope was found in this paper. More specifically, in the area of gentle slope (10°), the temperature difference increased with slope; in the area with slope between 10° and 40°, the temperature difference decreased with slope; and in the high‐slope area (> 40°), although the amount of data was small, temperature difference increased with slope (Xu et al., 2012). Slope affects the LST by changing the soil moisture content and determining the coverage of regional vegetation. In addition, in the mountainous areas, which have significant changes in elevation, the aspect directly affects the LST by influencing the exposure of the land surface to solar radiation. The relationship between temperature difference and aspect was very significant in this paper. The maximum and minimum values of temperature difference were distributed in the vicinities of the southern slope and northern slope, respectively. And the LST gradually increased with slope moving from north to south, which is consistent with the laws of natural geography. In urban areas, the effect of aspect on LST is much less significant that the effects of artificial surfaces and urban activities (Han et al., 2014).

As a surface coverage factor in this study, NDVI can regulate LST by altering the transpiration of plants. Some studies have reported negative correlations between LST and NDVI (Chen et al., 2006; Weng et al., 2004), whereas others have found only weak relationships because of differences in soil moisture, vegetation, and season (Cao et al., 2011; Sandholt et al., 2002). This study indicated a significant nonlinear correlation relationship between temperature difference and NDVI. For NDVI < 0.25, the temperature difference increased with increasing NDVI. In contrast, for NDVI > 0.25, the temperature difference decreased with increasing NDVI. Because of the relatively low degree of vegetation coverage in the study area, further studies are needed to definitively determine the relationship between LST and NDVI. The above results indicated that the effects of the different impact factors on temperature should be considered in the threshold study.

Many studies have shown that the ability to regulate temperature of a water body varies by season and time of day (Cao et al., 2011; Hathway & Sharples, 2012; Van Hove et al., 2015). The climate of the study area belongs to the dry‐hot valley climate. The dry season extends from November to April, when the reservoir is running at high head, the water level is approximately 600 m, and the reservoir area is large. During the wet season from May to October, the reservoir runs at low head, the reservoir water level is approximately 540 m, and the reservoir area is small. This paper was conducted during the dry season of the reservoir. The effect threshold of the reservoir on the surrounding temperature should be more obvious during the dry period than during the wet period. Furthermore, conducting the study during the dry season is beneficial for considering how to improve local climatic and soil conditions and protect plant and animal diversity (Zhang et al., 2005). The overall effect threshold of the reservoir on the temperature of the surrounding mountains was approximately 600 m. This value is significantly different that the threshold values reported for urban areas (e.g., 100 and 200 m reported by Li et al., 2008; Sun & Chen, 2012, respectively). This difference is attributed to the small sizes of the water bodies in urban areas along with the large number of high‐rise buildings, which hinder air flow and reduce the effect of water on the surrounding environment. The effect of water surface width on the effect threshold was also taken into account. In the reservoir head area (width ~1,000 m), the effect threshold was approximately 1,500 m. In contrast, in the tributary area (width ~60 m), the effect of the water on the surrounding temperature was almost negligible. Thus, the reservoir's effect on temperature was significantly affected by the water surface width. Future studies will evaluate the mechanism by which water surface width influences the surrounding temperature and explore the change in the effect threshold by season.

## 6 Conclusions

The temperature differences between various locations and the reservoir were calculated based on Landsat 8 TIRS data, and the most significant grid size was optimized using spatial analysis and partial correlation analysis. The partial correlation coefficient between temperature difference and Euclidean distance was determined for each buffer zone under the most significant grid size to evaluate the effect threshold of the reservoir on the temperature of the surrounding mountains. Finally, the effect of the water surface width on the temperature was considered. The results provide guidance for others to protect the animal and plant diversity of the area surrounding the reservoir and to responsibly exploit tourism resources. The results are summarized as follows:

1. The partial correlation coefficient in each buffer area at the grid size of 90 m is higher than those for other grid sizes; thus, 90 m is selected as the most significant grid size.
2. Under the most significant grid size of 90 m, the partial correlation coefficient in each buffer area decreases gradually with increasing distance from the reservoir. The effective effect threshold of the reservoir on the temperature of the surrounding mountains is approximately 600 m.
3. The relationship between water surface width and the effect threshold is significant. In the reservoir head area (water surface width ~1,000 m), the effect threshold is approximately 1,500 m. In contrast, the effect of the reservoir on the temperature of the surrounding mountains is negligible in the tributary area (water surface width ~60 m).

## Conflict of Interest

The authors declare no conflicts of interest relevant to this study. The founding sponsors had no role in the design of the study; in the collection, analyses, or interpretation of data; in the writing of the manuscript; and in the decision to publish the results.

## Supporting information

Data Set S1
